# Supplementary figures and images for: Efficacy of virtual reality balance training on rehabilitation outcomes following anterior cruciate ligament reconstruction: A systematic review and meta-analysis
Source: PLoS One. 2025 Jan 14;20(1):e0316400. doi: 10.1371/journal.pone.0316400 (PMC11731729; doi:10.1371/journal.pone.0316400)

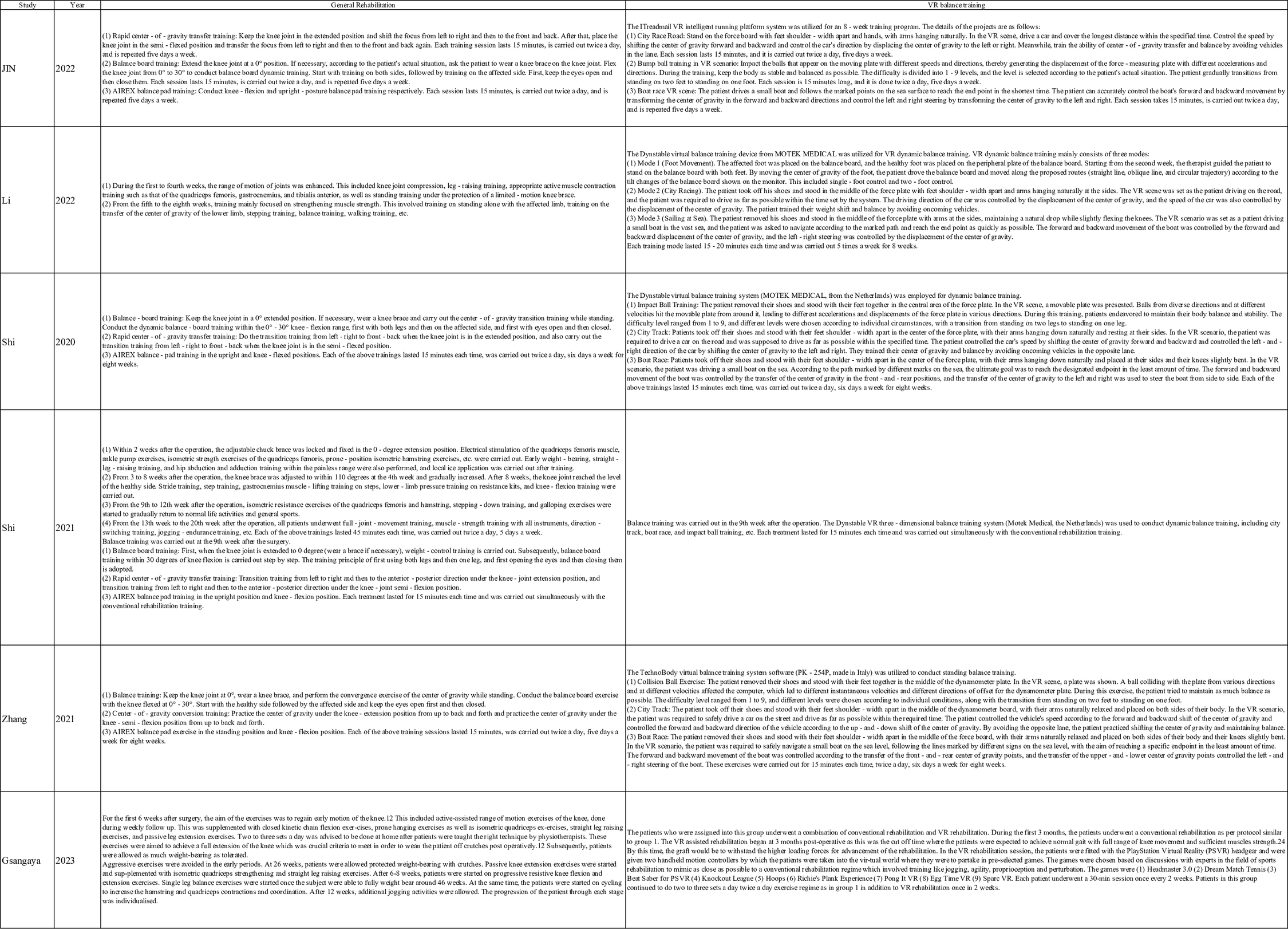

Supplement: S1 Table — (TIF) [file pone.0316400.s001.tif]

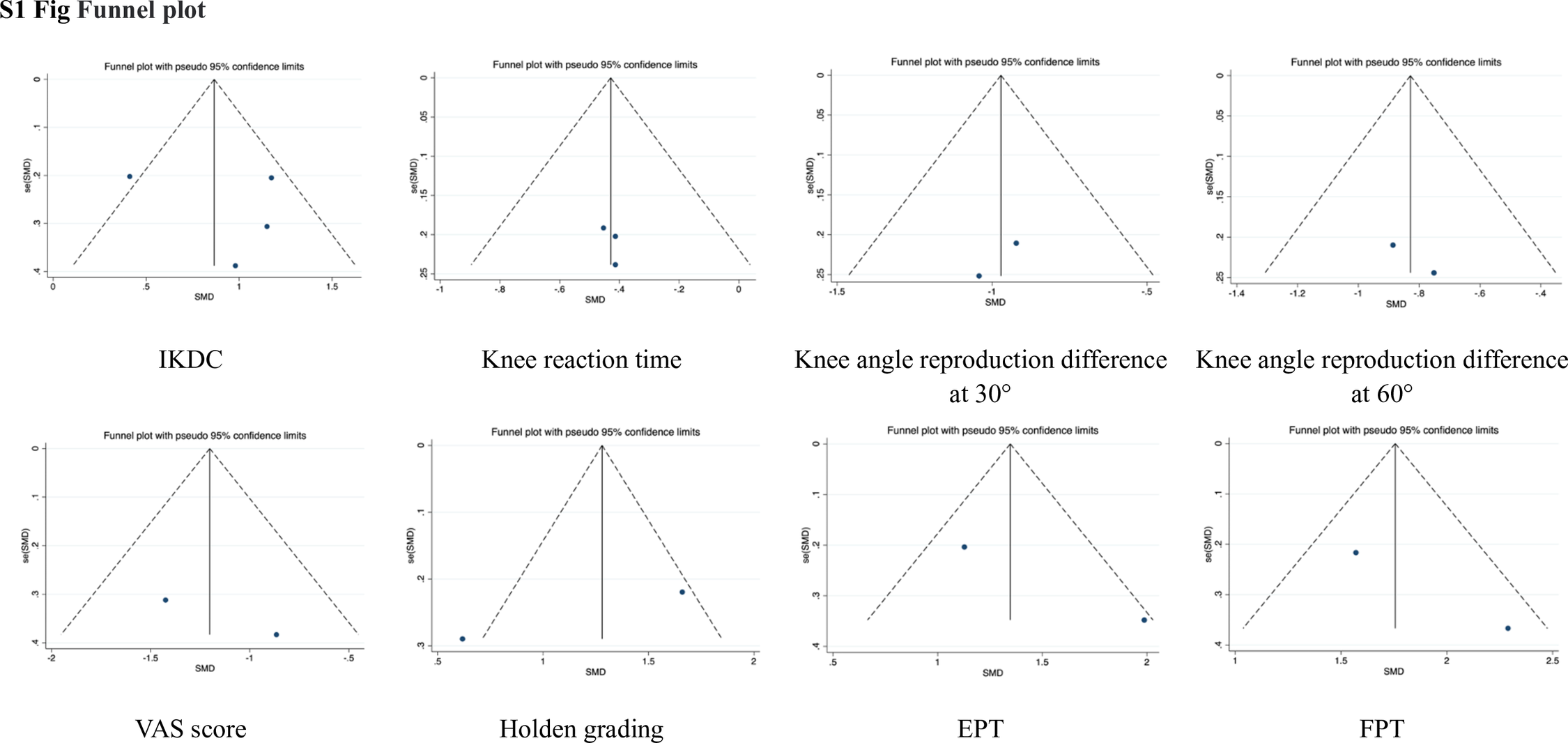

Supplement: S1 Fig — (TIF) [file pone.0316400.s002.tif]

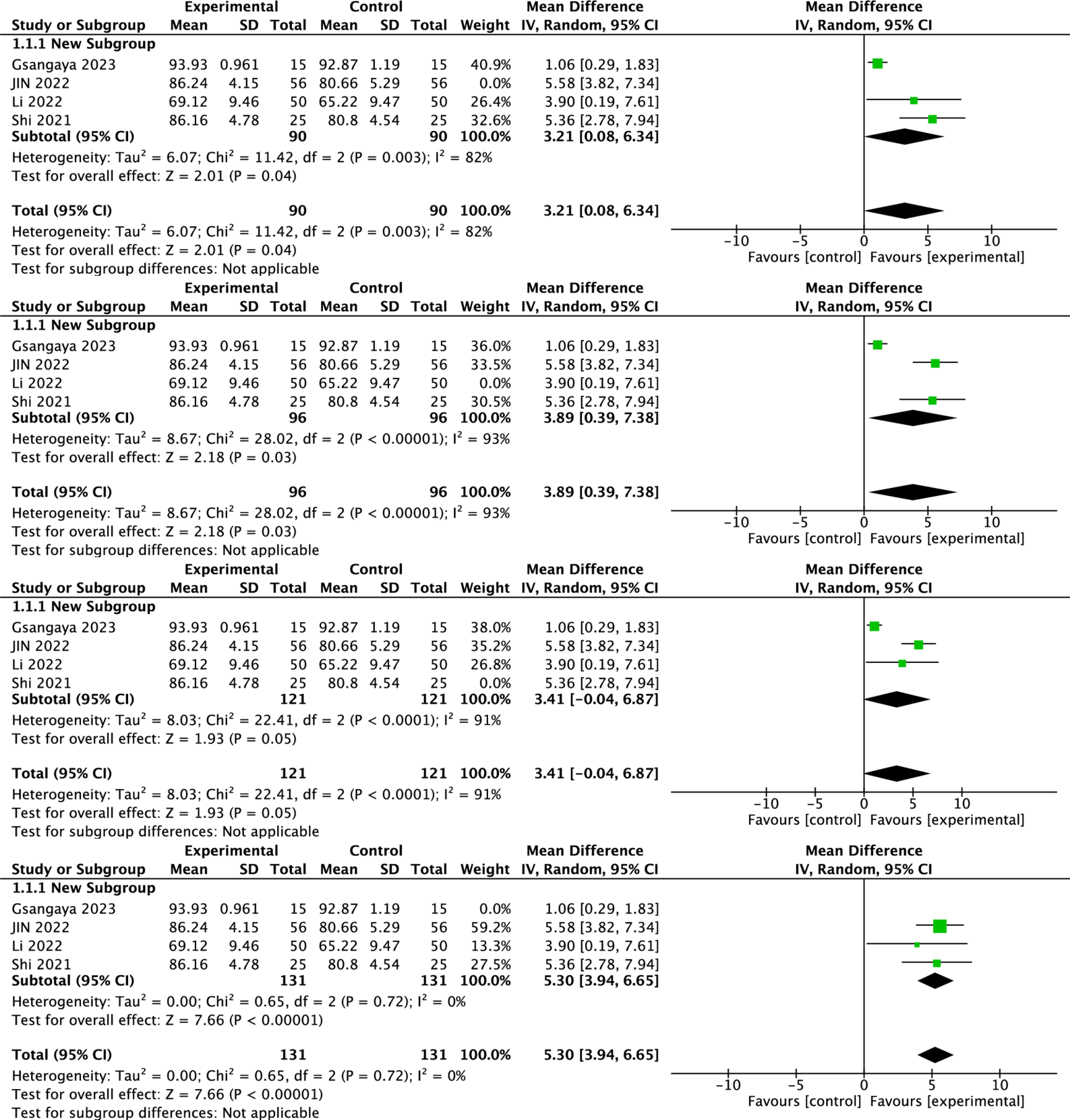

Supplement: S2 Fig — (TIF) [file pone.0316400.s003.tif]
