# Supplementary material for: Efficacy of virtual reality balance training on rehabilitation outcomes following anterior cruciate ligament reconstruction: A systematic review and meta-analysis
Source: PLoS One. 2025 Jan 14;20(1):e0316400. doi: 10.1371/journal.pone.0316400 (PMC11731729; doi:10.1371/journal.pone.0316400)
Supplement: S1 File — (DOCX) [file pone.0316400.s009.docx]

PubMed

(((((((((((((("Virtual Reality"[Mesh]) OR (Reality, Virtual[Title/Abstract])) OR (Virtual Reality, Educational[Title/Abstract])) OR (Educational Virtual Realities[Title/Abstract])) OR (Educational Virtual Reality[Title/Abstract])) OR (Reality, Educational Virtual[Title/Abstract])) OR (Virtual Realities, Educational[Title/Abstract])) OR (Virtual Reality, Instructional[Title/Abstract])) OR (Instructional Virtual Realities[Title/Abstract])) OR (Instructional Virtual Reality[Title/Abstract])) OR (Realities, Instructional Virtual[Title/Abstract])) OR (Reality, Instructional Virtual[Title/Abstract])) OR (Virtual Realities, Instructional[Title/Abstract])) OR (VR[Title/Abstract])) AND ((((((((((((((((((((((((((("Anterior Cruciate Ligament"[Mesh]) OR (Cruciate Ligament, Anterior[Title/Abstract])) OR (Anterior Cruciate Ligaments[Title/Abstract])) OR (Cruciate Ligaments, Anterior[Title/Abstract])) OR (Ligament, Anterior Cruciate[Title/Abstract])) OR (Ligaments, Anterior Cruciate[Title/Abstract])) OR (Anterior Cranial Cruciate Ligament[Title/Abstract])) OR (Cranial Cruciate Ligament[Title/Abstract])) OR (Cranial Cruciate Ligaments[Title/Abstract])) OR (Cruciate Ligament, Cranial[Title/Abstract])) OR (Cruciate Ligaments, Cranial[Title/Abstract])) OR (Ligament, Cranial Cruciate[Title/Abstract])) OR (Ligaments, Cranial Cruciate[Title/Abstract])) OR (ACL[Title/Abstract])) OR ("Anterior Cruciate Ligament Reconstruction"[Mesh])) OR ("Anterior Cruciate Ligament Injuries"[Mesh])) OR (ACL Injuries[Title/Abstract])) OR (ACL Injury[Title/Abstract])) OR (Injuries, ACL[Title/Abstract])) OR (Injury, ACL[Title/Abstract])) OR (Anterior Cruciate Ligament Injury[Title/Abstract])) OR (Anterior Cruciate Ligament Tear[Title/Abstract])) OR (ACL Tears[Title/Abstract])) OR (ACL Tear[Title/Abstract])) OR (Tear, ACL[Title/Abstract])) OR (Tears, ACL[Title/Abstract])) OR (Anterior Cruciate Ligament Tears[Title/Abstract]))

MEDLINE

( Virtual Reality OR Reality, Virtual OR Virtual Reality, Educational OR Educational Virtual Realities OR Educational Virtual Reality OR Reality, Educational Virtual OR Virtual Realities, Educational OR Virtual Reality, Instructional OR Instructional Virtual Realities OR Instructional Virtual Reality OR Realities, Instructional Virtual OR Reality, Instructional Virtual OR Virtual Realities, Instructional OR VR ) AND ( Anterior Cruciate Ligament OR Cruciate Ligament, Anterior OR Anterior Cruciate Ligaments OR Cruciate Ligaments, Anterior OR Ligament, Anterior Cruciate OR Ligaments, Anterior Cruciate OR Anterior Cranial Cruciate Ligament OR Cranial Cruciate Ligament OR Cranial Cruciate Ligaments OR Cruciate Ligament, Cranial OR Cruciate Ligaments, Cranial OR Ligament, Cranial Cruciate OR Ligaments, Cranial Cruciate OR ACL OR Anterior Cruciate Ligament Reconstruction OR Anterior Cruciate Ligament Injuries OR ACL Injuries OR ACL Injury OR Injuries, ACL OR Injury, ACL OR Anterior Cruciate Ligament Injury OR Anterior Cruciate Ligament Tear OR ACL Tears OR ACL Tear OR Tear, ACL OR Tears, ACL OR Anterior Cruciate Ligament Tears )

Cochrane Library

(Virtual Reality OR Reality, Virtual OR Virtual Reality, Educational OR Educational Virtual Realities OR Educational Virtual Reality OR Reality, Educational Virtual OR Virtual Realities, Educational OR Virtual Reality, Instructional OR Instructional Virtual Realities OR Instructional Virtual Reality OR Realities, Instructional Virtual OR Reality, Instructional Virtual OR Virtual Realities, Instructional OR VR):ti,ab,kw AND (Anterior Cruciate Ligament OR Cruciate Ligament, Anterior OR Anterior Cruciate Ligaments OR Cruciate Ligaments, Anterior OR Ligament, Anterior Cruciate OR Ligaments, Anterior Cruciate OR Anterior Cranial Cruciate Ligament OR Cranial Cruciate Ligament OR Cranial Cruciate Ligaments OR Cruciate Ligament, Cranial OR Cruciate Ligaments, Cranial OR Ligament, Cranial Cruciate OR Ligaments, Cranial Cruciate OR ACL OR Anterior Cruciate Ligament Reconstruction OR Anterior Cruciate Ligament Injuries OR ACL Injuries OR ACL Injury OR Injuries, ACL OR Injury, ACL OR Anterior Cruciate Ligament Injury OR Anterior Cruciate Ligament Tear OR ACL Tears OR ACL Tear OR Tear, ACL OR Tears, ACL OR Anterior Cruciate Ligament Tears):ti,ab,kw (Word variations have been searched)

Embase

('virtual reality':ti,ab,kw OR 'reality, virtual':ti,ab,kw OR 'virtual reality, educational':ti,ab,kw OR 'educational virtual realities':ti,ab,kw OR 'educational virtual reality':ti,ab,kw OR 'reality, educational virtual':ti,ab,kw OR 'virtual realities, educational':ti,ab,kw OR 'virtual reality, instructional':ti,ab,kw OR 'instructional virtual realities':ti,ab,kw OR 'instructional virtual reality':ti,ab,kw OR 'realities, instructional virtual':ti,ab,kw OR 'reality, instructional virtual':ti,ab,kw OR 'virtual realities, instructional':ti,ab,kw OR vr:ti,ab,kw OR 'virtual reality'/exp) AND ('cruciate ligament, anterior':ti,ab,kw OR 'anterior cruciate ligaments':ti,ab,kw OR 'cruciate ligaments, anterior':ti,ab,kw OR 'ligament, anterior cruciate':ti,ab,kw OR 'ligaments, anterior cruciate':ti,ab,kw OR 'anterior cranial cruciate ligament':ti,ab,kw OR 'cranial cruciate ligament rupture':ti,ab,kw OR 'cranial cruciate ligaments':ti,ab,kw OR 'cruciate ligament, cranial':ti,ab,kw OR 'cruciate ligaments, cranial':ti,ab,kw OR 'ligament, cranial cruciate':ti,ab,kw OR 'ligaments, cranial cruciate':ti,ab,kw OR 'anterior cruciate ligament reconstruction':ti,ab,kw OR 'anterior cruciate ligament injuries':ti,ab,kw OR 'acl injuries':ti,ab,kw OR 'acl injury':ti,ab,kw OR 'injuries, acl':ti,ab,kw OR 'injury, acl':ti,ab,kw OR 'anterior cruciate ligament injury':ti,ab,kw OR 'anterior cruciate ligament tear':ti,ab,kw OR 'acl tears':ti,ab,kw OR 'acl tear':ti,ab,kw OR 'tear, acl':ti,ab,kw OR 'tears, acl':ti,ab,kw OR 'anterior cruciate ligament tears':ti,ab,kw OR 'anterior cruciate ligament reconstruction'/exp)

Web of science

TS=(Anterior Cruciate Ligament OR Cruciate Ligament, Anterior OR Anterior Cruciate Ligaments OR Cruciate Ligaments, Anterior OR Ligament, Anterior Cruciate OR Ligaments, Anterior Cruciate OR Anterior Cranial Cruciate Ligament OR Cranial Cruciate Ligament OR Cranial Cruciate Ligaments OR Cruciate Ligament, Cranial OR Cruciate Ligaments, Cranial OR Ligament, Cranial Cruciate OR Ligaments, Cranial Cruciate OR Anterior Cruciate Ligament Reconstruction OR Anterior Cruciate Ligament Injuries OR ACL Injuries OR ACL Injury OR Injuries, ACL OR Injury, ACL OR Anterior Cruciate Ligament Inj**ury OR Anterior Cruciate** Ligament Tear OR ACL Tears OR ACL Tear OR Tear, ACL OR Tears, ACL OR Anterior Cruciate Ligament Tears )

AND

TS=(Virtual Reality OR Reality, Virtual OR Virtual Reality, Educational OR Educational Virtual Realities OR Educational Virtual Reality OR Reality, Educational Virtual OR Virtual Realities, Educational OR Virtual Reality, Instructional OR Instructional Virtual Realities OR Instructional Virtual Reality OR Realities, Instructional Virtual OR Reality, Instructional Virtual OR Virtual Realities, Instructional OR VR )
