# Supplementary material for: Efficacy of virtual reality balance training on rehabilitation outcomes following anterior cruciate ligament reconstruction: A systematic review and meta-analysis
Source: PLoS One. 2025 Jan 14;20(1):e0316400. doi: 10.1371/journal.pone.0316400 (PMC11731729; doi:10.1371/journal.pone.0316400)
Supplement: Attachment — Submitted filename: Response to reviewers1.docx [file pone.0316400.s010.docx]

[Aifeng Liu]

[The First Teaching Hospital of Tianjin University of Traditional Chinese Medicine]

[draifeng@163.com]

Editor,

PLOS ONE

Ref: Submission ID PONE-D-24-41399.

Dear Editor Khalid Taher Mohammed Al-Hussaini,

We sincerely appreciate the valuable feedback provided by both the reviewers and the Editor for our manuscript titled "Efficacy of virtual reality balance training on rehabilitation outcomes following anterior cruciate ligament reconstruction: A systematic review and meta-analysis " (Submission ID PONE-D-24-41399). We have thoroughly reviewed and carefully considered all the comments received, and we have made comprehensive revisions to the manuscript, with text changes highlighted in yellow, to enhance its quality and clarity. Please find below our point-by-point response to the comments received:

**Response to Editor (original comments by reviews are in blue color)**

****Editor:****

**1.Comment：**Please ensure that your manuscript meets PLOS ONE's style requirements, including those for file naming. The PLOS ONE style templates can be found at

https://journals.plos.org/plosone/s/file?id=wjVg/PLOSOne_formatting_sample_main_body.pdf and

https://journals.plos.org/plosone/s/file?id=ba62/PLOSOne_formatting_sample_title_authors_affiliations.pdf

**1. Reply：**Thank you for your comments. We have made a careful revision in line with the magazine's requirements.

**2.Comment：**As required by our policy on Data Availability, please ensure your manuscript or supplementary information includes the following:

A numbered table of all studies identified in the literature search, including those that were excluded from the analyses.

For every excluded study, the table should list the reason(s) for exclusion.

If any of the included studies are unpublished, include a link (URL) to the primary source or detailed information about how the content can be accessed.

A table of all data extracted from the primary research sources for the systematic review and/or meta-analysis. The table must include the following information for each study:

Name of data extractors and date of data extraction

Confirmation that the study was eligible to be included in the review.

All data extracted from each study for the reported systematic review and/or meta-analysis that would be needed to replicate your analyses.

If data or supporting information were obtained from another source (e.g. correspondence with the author of the original research article), please provide the source of data and dates on which the data/information were obtained by your research group.

If applicable for your analysis, a table showing the completed risk of bias and quality/certainty assessments for each study or outcome.  Please ensure this is provided for each domain or parameter assessed. For example, if you used the Cochrane risk-of-bias tool for randomized trials, provide answers to each of the signalling questions for each study. If you used GRADE to assess certainty of evidence, provide judgements about each of the quality of evidence factor. This should be provided for each outcome.

An explanation of how missing data were handled.

This information can be included in the main text, supplementary information, or relevant data repository. Please note that providing these underlying data is a requirement for publication in this journal, and if these data are not provided your manuscript might be rejected.

**2. Reply：**We have listed all the studies found in the literature search in a numerical table and also listed the reasons for exclusion. We name it "all data”. We have also listed all the data extracted from the main sources of the meta – analysis, as well as the complete bias - risk assessment table and evidence - quality - grade assessment table for each study. We have uploaded these data.

**3.Comment：**Thank you for stating the following financial disclosure:

"The funding support from the National Natural Science Foundation of China (Grant no. 81873316) and the Tianjin Health Commission Jinmen medical talent project (Grant no. TJSJMYXYC-D2-028) for this work is gratefully acknowledged."

Please state what role the funders took in the study.  If the funders had no role, please state: ""The funders had no role in study design, data collection and analysis, decision to publish, or preparation of the manuscript.""

If this statement is not correct you must amend it as needed.

Please include this amended Role of Funder statement in your cover letter; we will change the online submission form on your behalf.

**3. Reply：**We have changed the financial information: changing the National Natural Science Foundation of China (Grant no. 81873316) to the National Natural Science Foundation of China (Grant no. 82474538). The role played by the sponsor in this study has been pointed out in the author contributions. Please see line 385-386.

**4.Comment：**We note that your Data Availability Statement is currently as follows: All relevant data are within the manuscript and its Supporting Information files.

Please confirm at this time whether or not your submission contains all raw data required to replicate the results of your study. Authors must share the “minimal data set” for their submission. PLOS defines the minimal data set to consist of the data required to replicate all study findings reported in the article, as well as related metadata and methods (https://journals.plos.org/plosone/s/data-availability#loc-minimal-data-set-definition).

For example, authors should submit the following data:

- The values behind the means, standard deviations and other measures reported;

- The values used to build graphs;

- The points extracted from images for analysis.

Authors do not need to submit their entire data set if only a portion of the data was used in the reported study.

If your submission does not contain these data, please either upload them as Supporting Information files or deposit them to a stable, public repository and provide us with the relevant URLs, DOIs, or accession numbers. For a list of recommended repositories, please see https://journals.plos.org/plosone/s/recommended-repositories.

If there are ethical or legal restrictions on sharing a de-identified data set, please explain them in detail (e.g., data contain potentially sensitive information, data are owned by a third-party organization, etc.) and who has imposed them (e.g., an ethics committee). Please also provide contact information for a data access committee, ethics committee, or other institutional body to which data requests may be sent. If data are owned by a third party, please indicate how others may request data access.

**4. Reply：**The documents we submitted contain all the original data required for the replicated research results.

**5.Comment：**Please include your tables as part of your main manuscript and remove the individual files. Please note that supplementary tables (should remain/ be uploaded) as separate ""supporting information"" files

**5. Reply：**The tables has been inserted into the manuscript, and the supplementary tables is uploaded as a separate file.

**6.Comment：** Please include captions for your Supporting Information files at the end of your manuscript, and update any in-text citations to match accordingly. Please see our Supporting Information guidelines for more information: http://journals.plos.org/plosone/s/supporting-information.

**6. Reply：**We have added instructions for the Supporting Information files at the end of the manuscript and updated the corresponding text.

**Response to reviewers (original comments by reviews are in blue color)**

**Reviewer # 1:**

**1.****Comment：**The manuscript reports high heterogeneity in key outcomes such as the International Knee Documentation Committee (IKDC) score (I² = 90%). Although a sensitivity analysis was conducted, the sources of this heterogeneity are not adequately discussed. This raises concerns about the validity of the pooled results. High heterogeneity weakens the reliability of the findings and suggests that the included studies might differ significantly in their methods or patient characteristics. Further subgroup analyses or exploration of potential sources of heterogeneity (e.g., differences in rehabilitation duration or virtual reality training intensity) would strengthen the paper. Please provide a more thorough exploration of potential sources of variability (e.g., differences in intervention duration, VR systems used, patient characteristics). A deeper discussion of how the heterogeneity impacts the reliability of the meta-analysis results is required.

**1.****Reply：**We greatly appreciate your attention to detail. We further explored the IKDC result heterogeneity through subgroup analysis based on country, training frequency, and whether 3D was used in the intervention. The results revealed that, after grouping by country and training frequency, the within - group heterogeneity of each group was not significant. This indicates that the country and training frequency in the literature were among the sources of significant heterogeneity. When grouped by whether 3D was used, the within - group heterogeneity remained significant in the non - 3D group. Interestingly, though, the results in non - 3D group were not significantly different (MD = 3.41, 95%CI: -0.004~6.87, *P* < 0.0001, *I²* = 0%). In contrast, the 3D group showed a significant difference (MD = 5.36, 95%CI: 2.78~7.94, *P* < 0.0001). This suggests that 3D may not be the source of IKDC heterogeneity. Summary, the use of 3D may render the IKDC results of VR balance training significant. We supplemented the subgroup analysis with the images：

At the same time, we also added the sensitivity analysis pictures of IKDC and created the forest map after excluding each study as follows:

**2. Comment：**The GRADE approach reveals that many of the included outcomes are based on low or very low-quality evidence. The manuscript should emphasize the limitations this places on the conclusions. While the authors acknowledge the need for more high-quality studies, the current conclusions seem stronger than what the data supports.

**2.Reply：**When writing the manuscript, we reached the current conclusion because, although the quality of the evidence was low or very low, in the existing research, these results showed a certain consistent trend.

Thanks to your reminder, we also realize that although there is consistency in the results, due to the quality of evidence, our current conclusion may indeed seem stronger than what the data can support. When re - examining the manuscript, we found that during the writing process, we might have over - emphasized the universality of the results without fully considering the limitations of the evidence quality. We have clearly emphasized the influence of these limitations on the conclusion in the manuscript. It will be pointed out at the beginning of the conclusion part that, since many of the included results are based on low - or very - low - quality evidence, although the results show a certain trend, the conclusion has great uncertainty. We have already revised the conclusion. Please see line 372-378. We have highlighted the inclusion in yellow.
**3. Comment：**Consider revising the tone of the conclusions to reflect the low certainty of evidence more clearly. Thus, while the study attempts to draw conclusions about the benefits of virtual reality balance training after ACL reconstruction, the limitations in data quality, heterogeneity, and small sample size weaken the strength of the conclusions.

**3.Reply：**Thank you for your question. We have already revised the conclusion. Please see line 372-378. We have highlighted the inclusion in yellow.

**4. Comment：**The manuscript contains several grammatical errors and awkward phrasing. A professional language review is necessary.

**4.Reply：**Thank you for reading the entire text and pointing out our grammar mistakes. We have reviewed and corrected the entire text.

**5. Comment：**It is not clear what the authors consider as VR. Please elaborate on your definition of VR. For example, do you consider exergames as VR?

**5.Reply：**Thank you for your question. We believe that virtual reality is a means of providing rehabilitation for patients through human - computer interaction based on a variety of technologies and methods, and virtual reality games that are helpful for rehabilitation are also included. Please see line 81-85. We have highlighted the inclusion in yellow.

**6. Comment：**The details about the VR balance training interventions across studies are somewhat vague. Were the VR systems and training protocols comparable? More discussion on the variations in VR systems used (e.g., VR hardware, software, immersion level) would improve the interpretation of the findings.

**6.Reply：**We incorporated the details of VR balance training interventions in the included literature into S1 Table. We believe that the VR system and training protocols are comparable, because in the included studies, both the VR system and training protocols are balance training, and the purposes of their training actions are the same. Through literature review, we added details about the relevant VR system and increased the discussion. Please see line 299-308. We have highlighted the inclusion in yellow.

**7. Comment：**The presentation of tables and figures is generally clear, but some could benefit from more descriptive legends to help readers interpret the results without needing to refer back to the text.

**7.Reply：**Thank you for your comments. We have checked and revised all the charts in the full text for better reading.

**8. Comment：**Figure 6 is missing a caption

**8.Reply：**Thank you for your detailed review. It has been revised. We put it into S1 Fig.

**9. Comment：**Given the significant heterogeneity in some outcomes, have you considered conducting subgroup analyses (e.g., based on age, gender, or duration of intervention)?

**9.Reply：**Thank you for your question. We have discussed this issue, and we believe that the number of articles included in other highly heterogeneous results is too small for subgroup analysis to be meaningful.

**10. Comment：**How do you account for potential variations in the quality of virtual reality systems used across the studies? Could differences in the systems affect the outcomes?

**10.Reply：**Thank you for your comments. After careful discussion, we believe that the influence of the virtual reality system used in this study on patients is mainly reflected in the intervention duration and the degree of immersion. Since their specific rehabilitation methods are similar, we think that the potential differences in the quality of the virtual reality system are small. And for the intervention duration and the degree of immersion, we have all carried out subgroup analyses to determine the influence of their differences on the outcome. Please see line 243-254. We have highlighted the inclusion in yellow.

**11. Comment：**The GRADE assessment resulted in "very low" or "low" quality of evidence for most outcomes. How confident are you in the clinical relevance of the findings, and how would you suggest these results be used in practice?

**11.Reply：**We respectively examined the data and results of each paper. We think that although the level of evidence is relatively low, all the included references reported good results, that is, the application of VR has played a positive role in the rehabilitation of ACLR. Based on this, we believe that our results are meaningful. In practice, we suggest that VR balance training be carried out for ACLR patients when conditions permit, which is helpful for the recovery of knee joint function and proprioception of patients. More importantly, the use of VR will increase the fun and compliance, which we also mentioned in the discussion. Please see line 319-326. We have highlighted the inclusion in yellow.

**12. Comment：**Better explain how VR is used in ACLR rehabilitation. As you talk about the potential of VR to "revolutionize ACLR rehabilitation" please make clear how exactly VR support the rehabilitation process.

**12.Reply：**Thank you for your question. For the sake of more rigor, we have changed this sentence to: VR is increasingly being applied in a variety of rehabilitation fields, which shows the feasibility of VR. We have supplemented the applications of VR in other medical fields. Please see line 81-88. We have highlighted the inclusion in yellow. As for VR supporting the rehabilitation process, we added relevant content during the discussion. Please see line 319-326 and 349-351. We have highlighted the inclusion in yellow.

**13. Comment：**You intriduced the abbreviation VR in the introduction but cnstantly write virtual reality. keep it consistent.

**13.Reply：**Thank you for pointing out this problem, we have revised it.

**14. Comment：**“Despite ACLR being a common procedure, the rate of successful return to pre-injury functional status is not as high as desired, with only around 24% of patients achieving this outcome.” – The phrase "not as high as desired" is vague and not appropriate for a research paper.

**14.Reply：**We have changed the sentence you pointed out to "Although ACLR is a frequently - performed procedure, the proportion of patients who successfully return to their pre - injury functional state is only approximately 24% ".

**15. Comment：**"The intervention for the patients in the treatment group was combined with virtual reality balance training based on the control group". This is not clear.

**15.Reply：**It has been described in detail in S1 Table**.** Please see line 206.

**16. Comment：**More detailed descriptions of the virtual reality interventions used in the included studies would be helpful. For example, what specific types of virtual reality balance training were applied (e.g., immersive vs. non-immersive VR)

**16.Reply：**We have added the content you suggested to the discussion. Please see line 299-308. We have highlighted the inclusion in yellow.

**17. Comment：**What is knee reaction time? There is no reference for this parameter

**17.Reply：**Knee reaction time is evaluated through the Dynstable virtual balance assessment system (the Netherlands), which can reflect the response speed of the nervous system and the coordination ability of muscles.

**18. Comment：**What is routine rehabilitation (i.e. in table 1)? Be precise in describing the intervention for the CG.

**18.Reply：**It has been described in detail in S1 Table**.** Please see line 206.

**19. Comment：**The manuscript switches between fixed and random effects models depending on the level of heterogeneity, but the rationale for these choices is not fully explained. The choice to use fixed effects for outcomes with lower heterogeneity is valid, but more justification is needed for how these models were selected and how they influence the interpretation of the data.

**19.Reply：**Thank you for your question. We've had in - depth discussions on this issue. First, we reviewed the literature. Tufanaru's article suggested that for less than 5 literatures, the fixed effect model should be used, which is in line with our study's use of this model. Secondly, for the results with high heterogeneity, we still use the random - effects model more prudently. So, we cited Tufanaru's article in our study for evidence support. Please see line 187. We have highlighted the inclusion in yellow.

**20. Comment：**Although the manuscript correctly states that the small number of included studies limits the utility of the funnel plot and Egger's test for detecting publication bias, it still includes these analyses.

**20.Reply：**We have supplemented funnel plots for all outcome indicators. See S1 Fig. After our careful discussion, since the number of included literatures is less than 10 and some studies have only two literatures, we think that it is not very meaningful to conduct an Egger test, and it may be more appropriate for this article to detect publication bias by visually inspecting the funnel plot.

**21. Comment：**“Proprioception is transmitted by nerves and plays an important role in joint stability, posture, and motor control.” This sentence does not make sense.

**21.Reply：**Thank you for your comments. We have already deleted this sentence.

**22. Comment：**"In a study [38] in which virtual reality balance training improved peripheral nerve damage, it was found that the improvement in proprioception was most likely achieved due to visual activation in virtual reality." Your reference [38] does not state anything about improved nerve damage following VR balance training nor about improved proprioception. In addition, this study did not use VR, but simple visual biofeedback of ankle motion on a screen. Your interpretation of the findings from [38] are a very far fetch.

**22.Reply：**We greatly appreciate your attention to detail. We have replaced the references anew and modified the contents as follows: a pilot study [54] found that the immediate visual feedback provided by VR may facilitate joint realignment, thereby activating the proprioceptive sense responsible for joint position perception. Please see line 349-351. We have highlighted the inclusion in yellow.

**23. Comment：**“The exercise process included in it may reduce pain by raising the pain threshold or increasing endogenous paroxysmal substances, among other pathways.” – The term “endogenous paroxysmal substances” is unclear and is completely out of context.

**23.Reply：**Thank you for your comments. We have already changed "endogenous paroxysmal substances" to "reducing serum inflammation among other pathways" and revised the references. Please see line 330-331. We have highlighted the inclusion in yellow.

**24. Comment：**Location where items are reported in the text are not correct in the Prisma Checklist.

**24.Reply：**Thank you for your question. We have made some changes.

**25. Comment：**While the manuscript is generally intelligible, there are several grammatical issues and awkward phrasing that affect the clarity of the writing. Some examples include:
a) "The objective of this systematic review and meta-analysis is to clarify the effect the rehabilitation efficacy..." should be rephrased as "The objective of this systematic review and meta-analysis is to clarify the rehabilitation efficacy..."
b) "The prevalence of ACL injuries is relatively high among the younger population..." must be made more precise and concise.
c) “The IKDC score metrics were more heterogeneous, and a sensitivity analysis was performed on them, which revealed that the heterogeneity of the IKDC metrics was significantly reduced.” – The repetition of “IKDC metrics” and “heterogeneity” makes this sentence cumbersome.
d) “At the same time, due to the special physiological structure of women, the results may be different, so the gender should be distinguished in the design of the program and the differences in rehabilitation effects between different genders should be explored.” This sentence is cumbersome and unclear.
e) “[…] virtual reality balance training group rehabilitated a significant reduction in knee VAS scores, and the difference was statistically significant.”
f) “Controversial results were addressed by consulting with a third author when disagreements were encountered, they were discussed in consultation with a 3rd researcher (T.-C.G.).
These are just a few of the many grammatical and stylistic problems impair the readability and clarity of the text. To improve comprehensibility, a thorough language revision is required.

**25.Reply：**Thank you for your meticulous review of the article. All the above problems have been revised：

a) We have made the revisions according to your suggestions. Please see line 30-31. We have highlighted the inclusion in yellow.

b) We have made some revisions to this part. Please see line 62-63. We have highlighted the inclusion in yellow.

c) We have made some revisions to this part. Please see line 241-243. We have highlighted the inclusion in yellow.

d) After discussion, we decided that in order not to cause problems of understanding, this sentence has been deleted.

e) We have already revised this part as: This is consistent with the results of this study. Please see line 334-335. We have highlighted the inclusion in yellow.

f) We have already revised this part as: Any disagreements were resolved through a review of the full-text of the original articles by an additional author (T.-C.G.), followed by a discussion. Please see line 155-156. We have highlighted the inclusion in yellow.

We have reviewed and revised the full text.

****Reviewer 2:****

**1. Comment：**Title: report main findings in the title

**1.Reply：**Thank you for your comments. The research content and findings of this article center on the efficacy of anterior cruciate ligament rehabilitation. Given the numerous outcome indicators, which are not fully represented in the title, we have simplified the description of the results in the abstract for easier reading and comprehension.

**2. Comment：**abstract
any hypothesis?
what key words were used ?

**2.Reply：**By thinking about this study, We added hypotheses: We hypothesized that VR balance training would be more beneficial than conventional training for postoperative rehabilitation of ACLR patients. Please see line 101-103. We have highlighted the inclusion in yellow. We used the following keywords: Virtual reality; Anterior cruciate ligament reconstruction; Balance training; Meta-analysis; Randomized controlled trial.

**3. Comment：**conclusions: coehrent

**3.Reply：**Thank you for your comments. We carried out a subgroup analysis of the main outcome indicator, the IKDC score, and thus further revised the conclusion. Please find more details on lines 371-378 for the Conclusion. We have highlighted the inclusion in yellow.

**4. Comment：**introduction
too long and confusing
focus on your topic
report the necessity for this systematic review
report the rationale for your study
finish with aim and hypotheiss

**4.Reply：**Thank you for your comments. We revised the introduction as suggested by you and ended with aim and hypotheiss at the end. Please find more details on lines 61-103 for the introduction. We have highlighted the inclusion in yellow.

**5. Comment：**methods
very well reported

**5.Reply：**Thank you for your comments.

**6. Comment：**results
too long try to reduce

**6.Reply：**Thank you for your comments. We have simplified the description of the results. We added subgroup analysis of the main outcome indicators. Please look for more details from lines 243 – 254. We have highlighted the relevant part in yellow.

**7. Comment：**discussion
start with main findings of your paper
report what Is new
analyze current literature
improve limitations
highlight clinical impact of your study

**7.Reply：**Thank you for your comments. Revisions have been made as per your suggestions. First, we introduced the main findings of this article. See line 291-298. We have highlighted the relevant part in yellow. Subsequently, we reported on the wide application of virtual reality in the medical field at present. For more details, please see line 299-308. We discuss the results of this study in combination with the published articles. See line 309-361，We had a discussion about the limitations and the clinical impact of this study. See line 362-370. We have highlighted the relevant part in yellow.

**8. Comment：**conclusions
coherenet

**8.Reply：**Thank you for your comments. We carried out a subgroup analysis of the main outcome indicator, the IKDC score, and thus further revised the conclusion. Please see line lines 371-378 for the Conclusion. We have highlighted the relevant part in yellow.

**9. Comment：**references
add following
Loucas M, Loucas R, D'Ambrosi R, Hantes ME. Clinical and Radiological Outcomes of Anteromedial Portal Versus Transtibial Technique in ACL Reconstruction: A Systematic Review. Orthop J Sports Med. 2021 Jul 2;9(7):23259671211024591. doi: 10.1177/23259671211024591. PMID: 34277881; PMCID: PMC8255613.
D'Ambrosi R, Valli F, Di Feo F, Marchetti P, Ursino N. Use of tourniquet in anterior cruciate ligament reconstruction: Is it truly necessary? A prospective randomized clinical trial. J Orthop Surg (Hong Kong). 2024 Sep-Dec;32(3):10225536241293538. doi: 10.1177/10225536241293538. PMID: 39418227.
D'Ambrosi R, Carrozzo A, Meena A, Corona K, Yadav AK, Annibaldi A, Kambhampati SBS, Abermann E, Fink C. A slight degree of osteoarthritis appears to be present after anterior cruciate ligament reconstruction compared with contralateral healthy knees at a minimum of 20 years: A systematic review of the literature. J Exp Orthop. 2024 Apr 4;11(2):e12017. doi: 10.1002/jeo2.12017. PMID: 38577065; PMCID: PMC10993150.

**9.Reply：**Thank you for your comments. The references you provided make up for our shortcomings, and these references have been added to the article. Please see line 310 and line 367.

We are confident that these revisions have greatly improved the quality of the manuscript and brought it up to the standards of PLOS ONE.

Attached to this response letter is the updated manuscript, including all necessary changes. We believe these revisions effectively address the feedback received.

We would like to thank the reviewers and editors for their constructive feedback and guidance, which has been beneficial in refining our work.

We appreciate the opportunity to revise and resubmit the manuscript with hopefully positive results. We remain committed to addressing any further comments or suggestions from reviewers and editors. Thank you for your consideration.

Sincerely,

Aifeng Liu
